# Phylum Gemmatimonadota and Its Role in the Environment

**DOI:** 10.3390/microorganisms10010151

**Published:** 2022-01-12

**Authors:** Izabela Mujakić, Kasia Piwosz, Michal Koblížek

**Affiliations:** 1 Centre Algatech, Institute of Microbiology, Czech Academy of Sciences, Novohradská 237, 379 81 Třeboň, Czech Republic; mujakic@alga.cz (I.M.); kpiwosz@mir.gdynia.pl (K.P.); 2 Department of Ecosystem Biology, Faculty of Science, University of South Bohemia, Branišovská 1760, 37005 České Budějovice, Czech Republic; 3 National Marine Fisheries Research Institute, Kołłątaja 1, 81-332 Gdynia, Poland

**Keywords:** Gemmatimonadota, *Gemmatimonadetes*, anoxygenic photosynthesis, photosynthetic gene cluster, MAGs

## Abstract

Bacteria are an important part of every ecosystem that they inhabit on Earth. Environmental microbiologists usually focus on a few dominant bacterial groups, neglecting less abundant ones, which collectively make up most of the microbial diversity. One of such less-studied phyla is Gemmatimonadota. Currently, the phylum contains only six cultured species. However, data from culture-independent studies indicate that members of Gemmatimonadota are common in diverse habitats. They are abundant in soils, where they seem to be frequently associated with plants and the rhizosphere. Moreover, Gemmatimonadota were found in aquatic environments, such as freshwaters, wastewater treatment plants, biofilms, and sediments. An important discovery was the identification of purple bacterial reaction centers and anoxygenic photosynthesis in this phylum, genes for which were likely acquired via horizontal gene transfer. So far, the capacity for anoxygenic photosynthesis has been described for two cultured species: *Gemmatimonas phototrophica* and *Gemmatimonas groenlandica*. Moreover, analyses of metagenome-assembled genomes indicate that it is also common in uncultured lineages of Gemmatimonadota. This review summarizes the current knowledge about this understudied bacterial phylum with an emphasis on its environmental distribution.

## 1. Introduction

Bacteria are an important component of all ecosystems, playing key roles in microbial food webs and the biogeochemical cycles. Initially, knowledge about microorganisms originated from work on species that could be cultivated and characterized based on their morphology, cell structure, chemical composition, and metabolic activities. This has changed since progress in molecular methods has enabled the study of microorganisms in their natural environment without the need for cultivation [1,2]. Gene surveys and analyses of 16S rRNA genes from environmental samples allow detection and identification of in situ microbial diversity, and due to shotgun metagenomic sequencing, our knowledge on microbial diversity is continuously growing [3].

**Figure 1:**
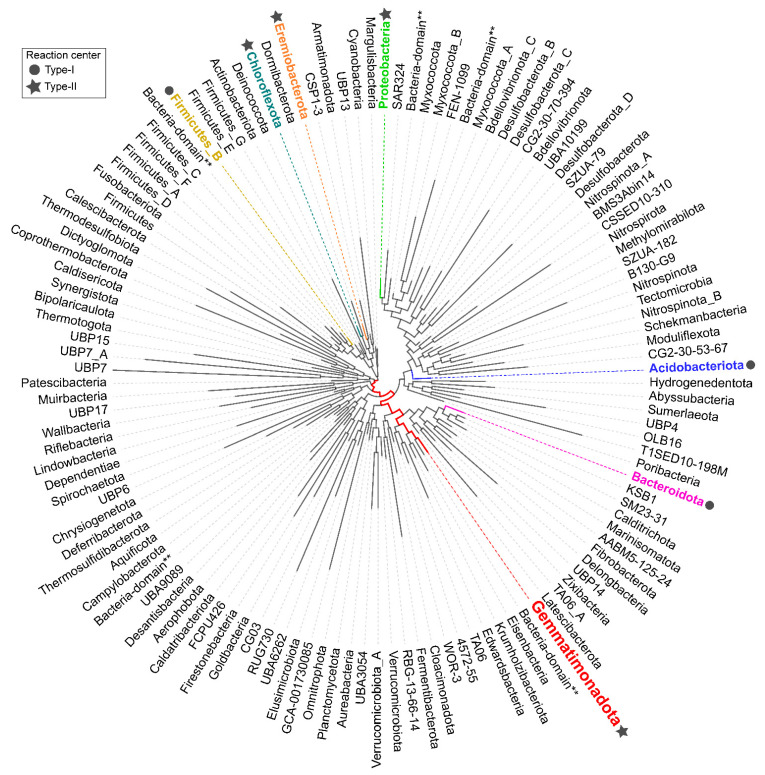
Phylogenomic tree of all bacterial phyla present in the Genome Taxonomy Database (GTDB) (Release RS95) [13,14] visualized using AnnoTree (version 1.2) [12], showing the position of Gemmatimonadota (marked in red). Additionally, along Gemmatimonadota, six other phyla that contain members capable of anoxygenic photosynthesis are marked in different colors, and the type of reaction center is indicated with a circle or a star (left legend). The double asterisk (**) means there is no corresponding taxonomy in GTDB for the genome so higher taxonomy level is used. The tree was edited in Inkscape (version 1.0).

One of the bacterial phyla established through molecular phylogenetic methods is Gemmatimonadota. It was first identified based on five environmental 16S rRNA gene sequences from deep-sea sediments, soils, and enhanced biological phosphorus removal (EBPR) reactor sludge, and it was named the BD-group [4,5,6]. Separately, based on the three 16S rRNA gene sequences from coastal sediment, it was called a candidate division KS-B [7]. It took only two years until a strain T27 belonging to the BD/KS-B group was isolated from a wastewater treatment plant in Japan. This organism was characterized and named *Gemmatimonas aurantiaca*, and the BD/KS-B group became a new phylum, Gemmatimonadota (initially named *Gemmatimonadetes* [8]). Phylogenetically, Gemmatimonadota are related with Fibrobacterota [9] and Chlorobi (Bacteroidota) [2,10,11]. Additionally, whole-genome comparison of all available bacterial phyla in the Genome Taxonomy Database (GTDB), visualized by AnnoTree [12], relates them with several newly proposed phyla, such as ‘*Candidatus* Eisenbacteria’, ‘*Candidatus* Krumholzibacteriota’, and ‘*Candidatus* Edwardsbacteria’ [13,14]. At present, there are six cultured species in the phylum, two of which are capable of anoxygenic photosynthesis [15,16]. Apart from Gemmatimonadota, anoxygenic photosynthesis is present in six other bacterial phyla and is scattered throughout the tree of life (Figure 1).

In contrast to the low number of described species, culture-independent methods indicate that members of Gemmatimonadota are ubiquitous and especially common in soils, limnic environments, and sediments [17]. Metagenomic analyses documented unexpected phylogenomic and metabolic diversity among uncultured Gemmatimonadota [18]. Moreover, several lineages of this phylum are capable of anoxygenic photosynthesis [15,16,19]. These results indicate that members of Gemmatimonadota likely play a specific role in the environment.

In this review, we summarize the current knowledge of culture and metagenomic studies on the ecology of Gemmatimonadota with a focus on their diversity and distribution in the environment.

## 2. Cultured Species

*Gemmatimonas aurantiaca* strain T-27^T^ was the first cultured Gemmatimonadota, which now represents the type species of class *Gemmatimonadetes,* genus *Gemmatimonas*. This aerobic chemoheterotrophic organism was isolated from an anaerobic–aerobic sequential batch reactor operated under enhanced EBPR conditions for wastewater treatment [8]. The other two members of the genus *Gemmatimonas* were isolated from freshwater habitats. A unique feature of these two organisms is the presence of bacteriochlorophyll-containing photosynthetic reaction centers [9,16]. Strain AP64^T^, isolated from the shallow desert lake Tiān é hú (Swan Lake) in North China, shares 96.1% sequence identity of the 16S rRNA gene with the type species *G. aurantiaca* T-27^T^. It is a facultative photoheterotroph, which requires organic substrates to grow, and the ability to harvest light provides an additional source of energy for its metabolism [20]. Based on its phototrophic lifestyle, strain AP64^T^ was named *Gemmatimonas phototrophica* sp. nov. [15]. Recently, the second photoheterotrophic strain TET16 was isolated from a stream in Northeast Greenland [16]. This strain represents a new species, as it has 95.7% sequence identity with the 16S rRNA gene of *G. phototrophica* and 95.9% identity with *G. aurantiaca*, and it was named *Gemmatimonas groenlandica* sp. nov. [16].

In contrast to the aquatic *Gemmatimonas* species, other cultured Gemmatimonadota originate from soils. A heterotrophic strain KBS708 was isolated from organically managed agricultural soil in Michigan USA. The strain with the tentative name ‘*Gemmatirosa kalamazoonensis*’ gen. nov., sp. nov. shares only 89% 16S rRNA gene sequence identity with *G. aurantiaca* [21]. However, this organism still awaits its valid description. Another soil species belonging to class *Gemmatimonadetes* was *Roseisolibacter agri* gen. nov., sp. nov. strain AW1220^T^, isolated from agricultural floodplain soil from Namibia [22]. Finally, *Longimicrobium terrae* strain CB-286315^T^ was isolated from a Mediterranean forest soil sample in Granada, Spain [23]. Since the new organism was relatively distant (16S rRNA identity 83–84%) from members of class *Gemmatimonadetes*, it established not only a new genus *Longimicrobium*, but also a novel class named *Longimicrobia* [23].

### 2.1. Physiology and Metabolism of Cultured Gemmatimonadota

All cultured Gemmatimonadota are chemoorganoheterotrophs, except for the facultative photoheterotrophic *G. phototrophica* and *G. groenlandica* [8,15,16,21,22,23]. They grow under aerobic or semiaerobic conditions, with temperature optima varying between 20 and 37 °C [16,22]. All species prefer low-salinity media—*G. aurantiaca* has the highest salinity tolerance of up to 0.8% NaCl [15]—as well as neutral or slightly alkaline pH. The only exception is ‘*Gemmatirosa kalamazoonensis*’, with a pH optimum between 5.5 and 6.5 [21]. Interestingly, *G. aurantiaca* possesses a metabolic potential for reduction of N_2_O, one of the strongest greenhouse gases [24,25], implying the possibly important ecological role of Gemmatimonadota in the environment.

**Figure 2:**
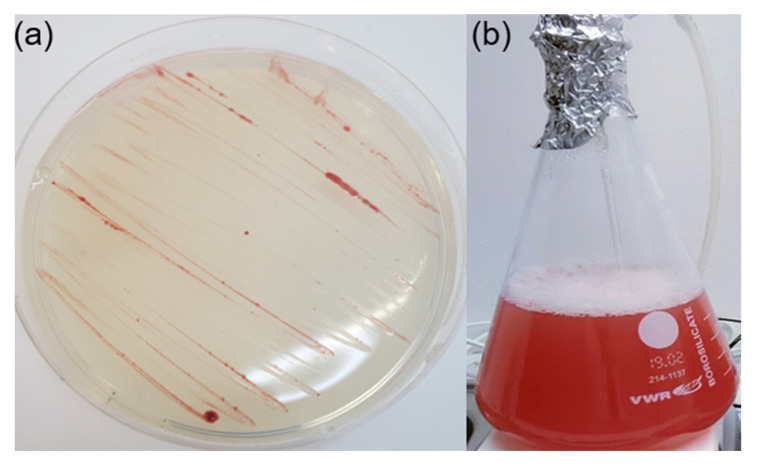
(**a**) Plates showing pure culture of *Gemmatimonas phototrophica* strain AP64 (**b**) and liquid culture of *Gemmatimonas groenlandica* strain TET16 (picture of TET16 is adapted from ref. [16]).

Gemmatimonadota cells are typically short rods, but occasionally, they can form over 10-µm-long filaments (not reported for *G. aurantiaca*). They divide by binary fission [8,15,16,21,22] and, except for the *L. terrae*, show budding morphology [23]. A typical trait of all cultured Gemmatimonadota is an intense pigmentation varying between orange and pink or even a reddish color in the stationary phase (Figure 2a,b). The predominant respiratory quinones are menaquinone-9 [8,21,22] or menaquinone-8 [15,16,23]. They are also naturally resistant to some antibiotics: *G. aurantiaca*, *G. phototrophica,* and ‘*Gemmatirosa kalamazoonensis*’ grow in the presence of ampicillin or penicillin [15,21], while *G. groenlandica* is resistant to bacitracin and chloramphenicol [16].

### 2.2. Anoxygenic Phototrophy in Gemmatimonadota

**Figure 3:**
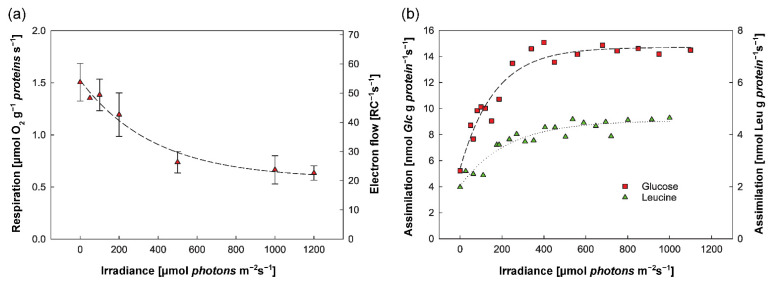
The figure shows (**a**) reduction of respiration in cells of *G. phototrophica* with increasing exposure to light and (**b**) increase in assimilation rates of ^3^H-glucose and ^3^H-leucine with exposure to light. The figure was adapted from ref. [20].

Gemmatimonadota, together with Firmicutes, Bacteroidota, Acidobacteriota, Proteobacteria, Chloroflexota, and *Candidatus* ‘Eremiobacteriota’, is one of few bacterial phyla capable of anoxygenic phototrophy [9,10,11] (Figure 1). Anoxygenic phototrophs can be distinguished based on the type of reaction center they have—either type I or type II, which differ with respect to the electron acceptors [26]. Both *G. phototrophica* and *G. groenlandica* contain type-II photosynthetic reaction centers [9,15,16]. They are facultative photoheterotrophs: They do not assimilate inorganic carbon and require a supply of organic substrates [9]. The ability of some members of Gemmatimonadota to harvest light provides them with additional energy, improves the efficiency of carbon utilization, and, consequently, increases their growth rate [20]. Light is used to generate ATP via photophosphorylation, which enables them to reduce respiration rate and, at the same time, increase assimilation rates of organic compounds, such as glucose and leucine (Figure 3a,b).

**Figure 4:**
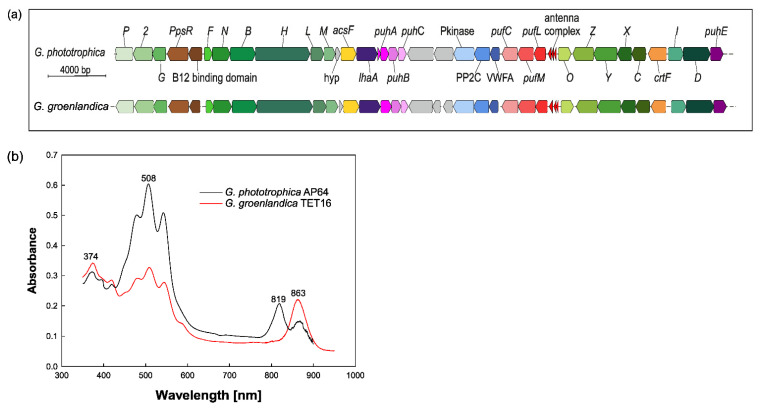
(**a**) Photosynthesis gene cluster of two cultured species of Gemmatimonadota, *G. phototrophica* and *G. groenlandica*. Different colors indicate genes involved in bacteriochlorophyll biosynthesis (green), carotenoid biosynthesis (orange), genes encoding the reaction center (*puf* operon-red), *puh* operon (pink/purple), other genes (brown and yellow), genes not involved in photosynthesis (blue), and hypothetical genes (gray). The figure was adapted from ref. [18]. (**b**) Absorption spectra of two cultures *G. phototrophica* (black) and *G. groenlandica* (red). In the near-infra-red range, *G. phototrophica* shows two peaks and *G. groenlandica* has only one. The figure was adapted from ref. [16].

Phototrophic Gemmatimonadota organize their genes involved in bacteriochlorophyll biosynthesis, expression and assembly of the photosystem, carotenoid biosynthesis, and regulatory functions in a photosynthesis gene cluster (PGC) [9]. The PGCs in *G. phototrophica* and *G. groenlandica* share the same synteny with two big superoperons (Figure 4a) and high DNA sequence identities (70–100%) [16]. Based on this organization, which is also common among phototrophic Proteobacteria [27], and the close phylogenetic relationship, it was suggested the source of the PGC for Gemmatimonadota was a horizontal gene transfer (HGT) event from Proteobacteria, specifically Gammaproteobacteria [9]. Intra-phylum HGT events are common in phototrophs and were reported for Cyanobacteria, Proteobacteria, and Chloroflexota [11,27,28,29,30,31]. The HGT of the complete PGC was documented for *Rhodobacteraceae* (Alphaproteobacteria) [32]. However, *G. phototrophica* represents the first known HGT event of a complete PGC between phototrophic and non-phototrophic representatives of different phyla [9,11,33].

The photosynthetic reaction centers of *G. phototrophica* are complexed with a unique double-concentric light-harvesting system, which manifests in two infra-red absorption bands [34] (Figure 4b). *G. phototrophica* synthesizes bacteriochlorophyll *a* constitutively. The photosynthetic apparatus was expressed even in cultures grown for one year under continuous darkness [15] and, in contrast to proteobacterial aerobic anoxygenic phototrophs [35], bacteriochlorophyll *a* is also produced under continuous light [20]. *G. phototrophica* contains over 10 different carotenoids [9], most still uncharacterized, which give the cells a specific red to pink color. Two identified carotenoids, also found in the heterotrophic member *G. aurantiaca,* are oscillol 2-rhamnoside and oscillol 2,2′-dirhamnoside [9,36]. They are believed to protect the cells from reactive oxygen species and excess light [9,37]. Recently, a unique carotenoid that is present only in the photosynthetic complex of *G. phototrophica* was described and named gemmatoxanthin [38]. Only limited information is available about the second phototrophic Gemmatimonadota *G. groenlandica*. It contains a slightly different set of carotenoids. Interestingly, despite the highly similar PGCs, *G. groenlandica* shows just one broad band in the absorption spectrum (Figure 4b), which may suggest a different organization of its light-harvesting antenna [16].

## 3. Environmental Distribution

High-throughput sequencing of environmental 16S rRNA genes documented that the phylum Gemmatimonadota is cosmopolitan, with members distributed across a wide range of natural environments. They are found in soils [39,40,41,42], permafrost [43,44,45,46], rhizospheres [47,48,49,50,51,52], freshwater lakes and sediments [53,54,55,56,57,58], activated sludge [8,59,60], deep-sea sediments [61,62,63,64], marine sponge symbionts [65,66,67,68], and a brackish estuary [69].

**Figure 5:**
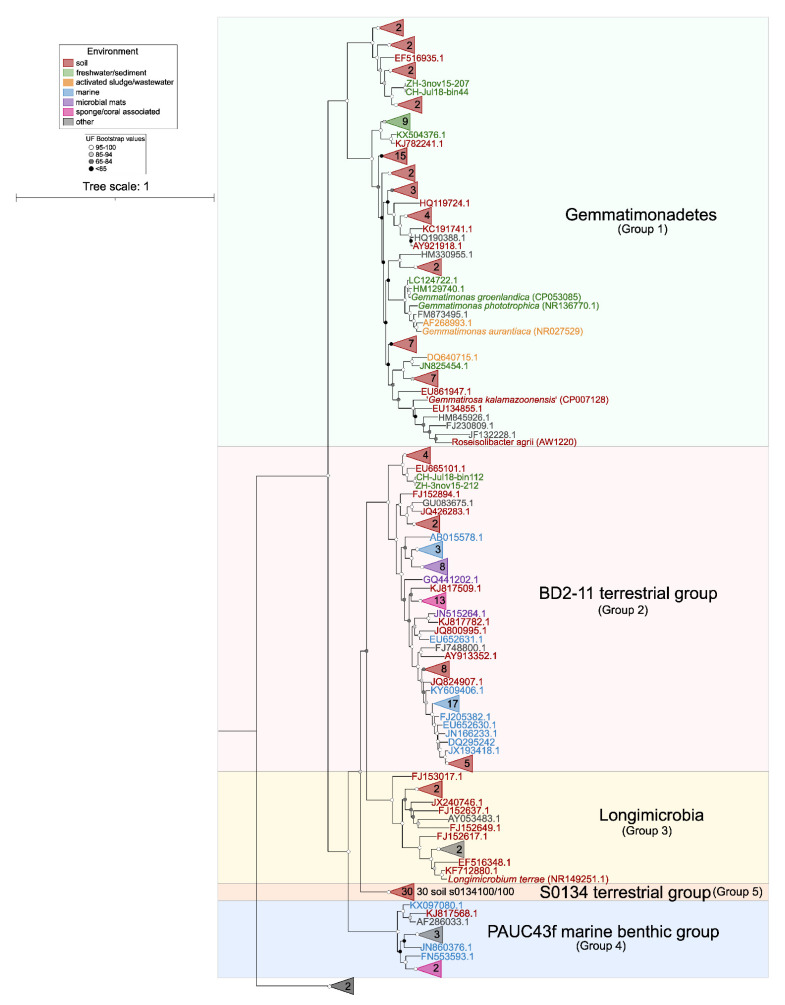
Maximum-likelihood phylogeny of 16S rRNA genes of Gemmatimonadota sequences recovered from the SILVA SSU v138 database (in total, 169 sequences, >1200 bp, sequence quality >94%, downloaded on 23 November 2021), 36 16S rRNA gene sequences whose accession numbers were taken from [22,59], and 12 sequences taken from [18]. All of the accession numbers are provided as Supplement Table S1. The phylogenetic tree was made with IQ-TREE [72,73], the TIM3 + F + I + G4 substitution model (chosen as the best-fitting model by ModelFinder [74]), and 1000 ultrafast bootstrap replicates. The sequences are colored based on the environment from which they originate (left legend). Numbers at collapsed branches indicate the number of sequences. All five class-level groups are indicated through vertical delimiters in the right part of the figure. The strength of support for internal nodes (assessed by ultrafast bootstrapping) is shown through gray-scale circles (left legend). Two sequences belonging to Fibrobacterota (*Fibrobacter succinogenes* and *Fibrobacter intestinalis*) were used as an outgroup. The phylogenetic tree was visualized using iTOL [75] and edited in Inkscape (version 1.0).

The increasing number of environmental sequences has revealed high uncultured diversity within Gemmatimonadota. In 2014, Hanada and Sekiguchi proposed its phylogeny encompassing both cultured species and environmental sequences, dividing the phylum into five class-level lineages (Figure 5). Group 1, which corresponds to the class *Gemmatimonadetes*, was the most represented, with most sequences originating from soils and a high proportion from activated sludge in wastewater treatment plants and freshwaters. Group 2, also referred to as the BD2-11 terrestrial group, contains sequences from marine sediments and sponges in addition to those from soils [59]. Group 3 is an equivalent to class *Longimicrobia* and includes the species *Longimicrobium terrae* [23], as well as sequences from soils and other environments (oil field, gas hydrate). Group 4, also referred to as the PAUC43f marine benthic group [70], contains sequences from marine sediments and sponges [59]. Finally, Group 5, or the S0134 terrestrial group [71], contains environmental sequences with terrestrial origin, mostly from different types of soil.

**Figure 6:**
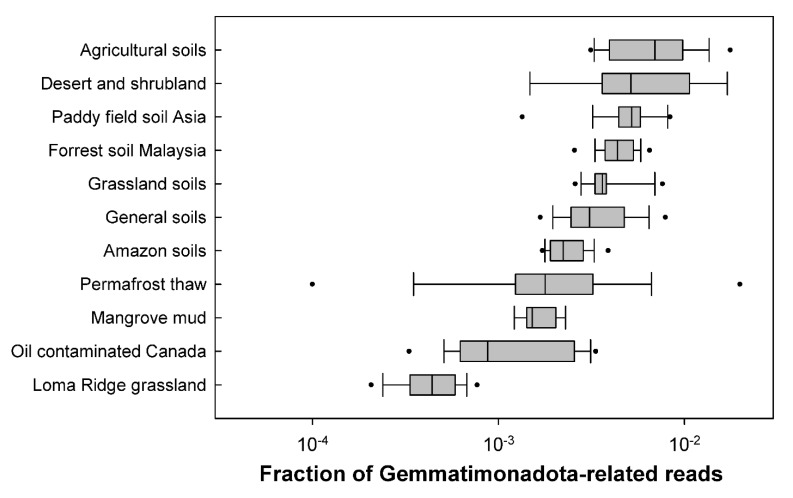
Gemmatimonadota-related reads recovered from different types of soils. The figure is based on metagenomic survey data published in [17].

A global picture of the distribution of Gemmatimonadota in various environments was obtained by a massive search in over 30,000 metagenomes that were publicly available at that time [17]. One thousand seven hundred and six metagenomes contained sequences related to Gemmatimonadota, which were most abundant in soils, with the largest proportion (0.3–1.8%) being found in agricultural soils (Figure 6). Gemmatimonadota were also abundant in wastewater treatment, biofilms, and plant-associated habitats, while smaller numbers were found in aquatic environments, such as lakes, rivers, estuaries, and springs.

### 3.1. Distribution in Soils

Gemmatimonadota are the eighth most abundant bacterial phylum in soils, accounting for about 1–2% of soil bacteria worldwide [41]. From the complete top-ten list (Proteobacteria, Actinobacteriota, Acidobacteriota, Planctomycetota, Chloroflexota, Verrucomicrobiota, Bacteroidota, Gemmatimonadota, Firmicutes, and Armatimonadota), Gemmatimonadota and Armatimonadota are plausibly the least-studied groups. A similar picture emerged from a study on the biogeographic distribution of Gemmatimonadota based on an analysis of sequences of 16S rRNA gene available in public databases [40]. They showed that, although they are present in sediments and other environments, the maximum number of sequences came from different types of soils, including grassland, agricultural, forest, or contaminated soils. The cosmopolitan distribution of Gemmatimonadota in various soils suggests that they are generalist species with a versatile metabolism that is able to adapt to a wide range of nutrients.

A unique environment where Gemmatimonadota have been found is in the glacier forefields of East Antarctica [76] and the McMurdo Dry Valleys, Antarctica. The soils of McMurdo Dry Valleys, originally thought to be sterile or to have low diversity of microorganisms [46], are dominated by Actinobacteriota, Acidobacteriota, Gemmatimonadota, and Bacteroidota [43,77]. Gemmatimonadota were suggested to be adapted to dry environments because they occur in high relative proportions in semiarid and arid soils and deserts [46,78,79,80,81,82]. Moreover, they were shown to be positively influenced by rainfall reduction and to be more abundant during drought [42]. Finally, even in soil aggregates, their relative abundance is higher in dry and semi-aerobic inner parts of microaggregates [83].

The pH is another factor that influences the abundance of Gemmatimonadota in soils, and it seems that they prefer neutral pH over acidic pH [40,84,85]. Interestingly, Gemmatimonadota dominated in alkaline [86] and highly saline soils and represented almost 17% of all bacterial reads [87].

Gemmatimonadota were also found to be one of the bacterial phyla that are positively correlated with vegetation restoration. As one of top-ten most abundant phyla that strongly increased with revegetation, their relative abundances were above 2% [88] and were positively correlated with plant richness and soil nutrients such as carbon. Moreover, they were one of seven more dominant bacterial phyla, with an abundance above 1%, which positively correlated with total carbon, nitrogen, and phosphorus in soil [89]. These studies showed the influence of high nutrient concentration on the abundance of Gemmatimonadota and their possible key role in soil ecosystems [88,89].

### 3.2. Distribution in Aquatic Habitats

Several reports noted the presence of Gemmatimonadota in freshwater lakes and sediments [19,53,54,55,56,90] or estuaries [69,91], and their sequences from public metagenomes were identified in lakes, rivers, estuaries, and marine ecosystems [17].

**Figure 7:**
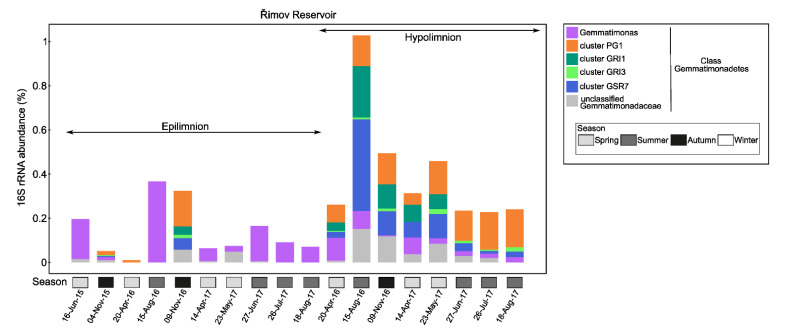
The relative abundances of 16S rRNA gene of Gemmatimonadota from metagenomes of a freshwater Římov Reservoir in the Czech Republic. Only the class *Gemmatimonadetes* is represented in Římov Reservoir, and newly defined clusters are shown (legend on the right). On the *x*-axis are shown sample dates and the season (gray-colored boxes), and on the *y*-axis is shown the percentage of Gemmatimonadota in the prokaryotic community. The figure was adapted from ref. [18].

The analysis of the microbial community in the stratified, warm, monomictic, freshwater Grand Lake, OK, USA showed that Gemmatimonadota, mostly genus *Gemmatimonas*, had higher relative abundance in September in the hypolimnion of the lake, where the oxygen had lower concentrations due to the sedimentation of organic matter, while in March and June, they were rare [92]. Their average abundance was around 1%. In a metagenomic study of Lake Baikal, two novel metagenome-assembled genomes (MAGs) of Gemmatimonadota were reported, one closely related to *G. phototrophica*, which showed higher abundance at 20 m, and the other more similar to the soil representative ‘*Gemmatirosa kalamazoonensis*’, with low abundances at both 5 and 20 m depth [93]. Until recently, it was not clear whether the Gemmatimonadota reported in freshwater environments were limnic species or they originated from surrounding soils. The limnic nature of this group was shown in a large study of several freshwater metagenomes from five freshwater lakes. The lakes differed in trophic status, and the presence of Gemmatimonadota was documented over the whole year at different depths in the lakes—both epilimnion and hypolimnion [18]. Their relative abundance based on the 16S rRNA gene ranged from 0.02 to 0.6% of total bacteria in epilimnion and up to 1% in hypolimnion. Genus *Gemmatimonas* occurred at both depths, but was more abundant in epilimnion. From these freshwater metagenomes, 45 MAGs of Gemmatimonadota were assembled, showing their great diversity in freshwaters [18]. Moreover, several novel genus-level clusters were proposed [18], including a newly defined photoheterotrophic cluster PG1 that is present in the hypolimnion (Figure 7).

**Figure 8:**
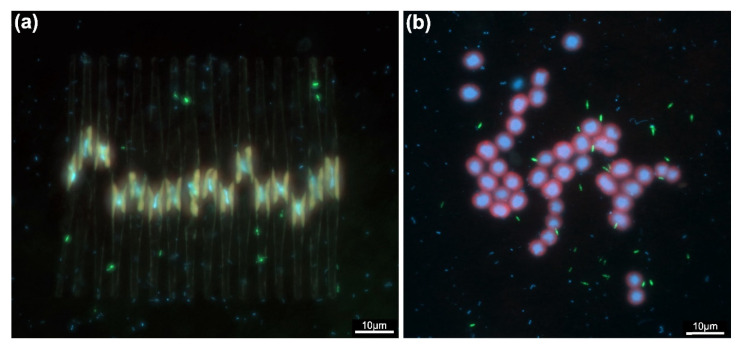
CARD-FISH images showing association of *Gemmatimonadetes* cells with colony of (**a**) *Fragilaria* sp. and (**b**) *Microcystis* sp. The signal of the probe is shown in green, DAPI staining in blue and autofluorescence in red. The photographs were taken by dr. Tanja Shabarova from Institute of Hydrobiology, Biology Centre of the Czech Academy of Sciences. Figure was adapted from ref. [18].

Different cell morphologies, depending on the depth, were seen in samples from the meso-eutrophic Římov Reservoir in the Czech Republic by using catalyzed-reported deposition–fluorescence in situ hybridization (CARD-FISH). Small, free-living cells were present in the hypolimnion, whereas in the epilimnion, cells were larger and were found in association with diatoms (*Fragilaria* sp.) and cyanobacteria (*Microcystis* sp.) (Figure 8a,b) [18]. Gemmatimonadota could benefit in such a co-occurrence by obtaining organic carbon and, in return, providing inorganic nutrients [94]. Additionally, this dependence was suggested as one of the possible reasons for the low number of cultured members of this phylum. Further analyses of the metabolism of Gemmatimonadota are needed to confirm this relationship.

Sequences from BD2-11 terrestrial group, were reported in sediments of Siberian soda lakes [95]. In addition, MAGs containing phototrophic genes were recovered in soda lake sediments, including one that also contained genes encoding large subunit of RuBisCo [19]. Furthermore, in sediment samples of shallow hypersaline Tuz lake in Turkey, Gemmatimonadota represented 2.7% of total 16S rRNA gene bacterial reads [96].

In marine environments, Gemmatimonadota seems to be mostly associated with sediments [97,98], deep-sea hydrothermal vents [64,99,100], and sponges [101,102,103]. In the deep-ocean sediment habitats of the Mariana and Massau trenches in the Pacific Ocean, the class *Gemmatimonadetes* was one of the dominant groups in the active bacterial community (in rRNA libraries), with an average relative abundance of OTUs of 13.30% and 9.93%, respectively [104]. *Gemmatimonadetes* were suggested to be a keystone group playing an important role in cycling of organic carbon due to their metabolic strategies [104]. In sediments of the South Eastern Arabian Sea, they were also abundant and represented 2.4% of the total bacterial 16S rRNA reads [105].

**Figure 9:**
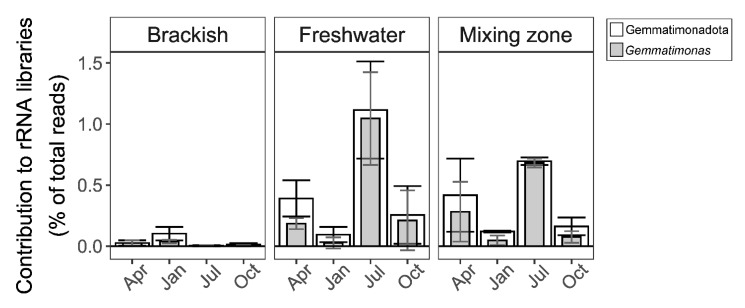
Gemmatimonadota rRNA reads recorded in the Gulf of Gdańsk (Baltic Sea) and the estuary of the Vistula River. The figure shows the percentage of reads associated with Gemmatimonadota (white), as well as the genus *Gemmatimonas* (gray). The salinity of brackish waters was ≥7‰, that of freshwater was <0.5‰, and that of the mixing zone was about 3.5‰. The data used to generate the figure were published in [69].

Finally, active Gemmatimonadota were found in brackish water in the Gulf of Gdańsk (Baltic Sea) at the estuary of the Vistula River [69]. The highest contribution of Gemmatimonadota to rRNA-based amplicon libraries was observed in summer in a river (>1.1%) and in the mixing zone at a salinity of 3.5‰ (Figure 9). The genus *Gemmatimonas* dominated in these habitats, especially in summer, when it made up over 90% of all Gemmatimonadota reads. Both the phylum Gemmatimonadota and genus *Gemmatimonas* were less active (<0.1% and <0.04% of reads, respectively) in the brackish waters of the Baltic Sea (salinity above 7‰). Interestingly, Gemmatimonadota were not reported at all from the DNA-based surveys of open Baltic Sea waters [106,107].

### 3.3. Other Environments

Studies of Gemmatimonadota in other environments are scarce. However, they seem to be very important members of bacterial communities in activated sludge and different wastewater treatments. The first cultured species, *G. aurantiaca,* was isolated from activated sludge [8], and higher proportions of both heterotrophic and photoheterotrophic Gemmatimonadota were detected in wastewater metagenomes [17]. In batch reactors used for pretreatment of urea wastewater, Gemmatimonadota became the dominant group and increased their relative abundance to over 50%, exceeding that of even Proteobacteria [108]. It seems that this group could be connected to intracellular urea hydrolysis [109], and urea could be used as an energy source and an important substrate [108]. Gemmatimonadota were also abundant in aquaculture wastewater and in soil irrigated with this water [110], an outlet of wastewater generated during nitrocellulose production [111], and wastewater treatment plants with high salinity [112].

Biofilms and microbial mats are yet another environment with a noticeable presence of Gemmatimonadota [17,113]. They were part of bacterial communities that formed the base of biofilms attached to a substrate as opposed to streamer structures that floated in water [114] and part of communities of microplastic biofilms [115]. In hypersaline microbial mats under different tidal activity, Gemmatimonadota were the most active in autumn, yet showed high relative RNA proportions in all seasons in tidal mats characterized by dominance of diatoms and the influence of currents and waves [116].

### 3.4. Distribution of Phototrophic Gemmatimonadota

The distribution and diversity of anoxygenic phototrophs is frequently detected using specific photosynthetic genes serving as molecular markers. The most common molecular marker for phototrophic Proteobacteria, Chloroflexota, and Gemmatimonadota is the *pufM* gene encoding the M subunit of the bacterial type-II reaction center. A more universal marker is gene *bchY* encoding the chlorophyllide reductase subunit Y, since it targets all anoxygenic phototrophic species [117]. However, this marker is not suitable for phototrophic Gemmatimonadota because of its high similarity to the sequences of Proteobacteria. To avoid this, another marker, gene *acsF*, which encodes aerobic oxidative cyclase in many phototrophic organisms, was introduced in a study of the freshwater Lake Taihu [118]. The previous studies documented that the *acsF* gene marker can reliably differentiate between phototrophic Proteobacteria, Gemmatimonadota, and Cyanobacteria [9,118,119]. Amplification of the *acsF* genes from Lake Taihu samples showed that phototrophic Gemmatimonadota represented 17.3% of the *acsF* reads in deep samples and 10.5% in the shallow-sediment samples, while in the water column, they represented only 0.67% of the reads [118].

*AcsF* sequences from phototrophic Gemmatimonadota were found in 161 metagenomes from different environments, such as wastewater treatment plants, soils, lake water columns and sediments, estuarine waters, biofilms, and plant-associated habitats. However, no *acsF* sequences were found in marine waters [17]. This increased contribution in non-marine aquatic environments suggests that phototrophic Gemmatimonadota may prefer different habitats from those of non-phototrophic species. The diversity of uncultured phototrophic Gemmatimonadota seems to be comparable to the diversity of Proteobacteria [17]. Photoheterotrophic members were also shown to express the photosynthetic genes in freshwater environments. In a study of two lakes in the Czech Republic, the relative abundance of *pufM* from Gemmatimonadota in the libraries prepared from RNA exceeded that from DNA libraries, indicating that photoheterotrophic Gemmatimonadota were active members of planktonic communities of anoxygenic phototrophic bacteria [120].

Although only two cultured photoheterotrophic species are available so far, a recent metagenomic study reported a high diversity of photoheterotrophic Gemmatimonadota in freshwater lakes [18]. They recovered 19 MAGs belonging to different genera that contained PGCs with similar organizations of genes to those of *G. phototrophica* and *G. groenlandica* [16], which indicates that phototrophic genes are conserved in these phyla. The abundance of phototrophic MAGs varied over the seasons, and they were present in both the epilimnion and hypolimnion [18]. MAGs of phototrophic Gemmatimonadota have also been reported in deep layers of Lake Baikal [93] and sediments of a soda lakes [95,121]. Interestingly, while some photoheterotrophic MAGs recovered from freshwater lakes containedRuBisCO-like protein (RLP) [18], which is considered as only a homologue of RuBisCO without carboxylation activity (type IV RuBisCO) [122,123], MAGs recovered in a soda lake showed the presence of genes encoding a large subunit of the RuBisCO enzyme. Moreover, six MAGs from these soda lakes contained all genes involved in the Calvin cycle [121]. This suggests that they represent the first photoautotrophic Gemmatimonadota, expanding the list of roles that these bacteria play in the environment.

## 4. Summary and Perspective

Members of Gemmatimonadota are present in many different environments. At the moment, most of the information is available from soils [39,40,41], and recently also from freshwater lakes [18,92,120]. Future research should also focus on other environments, such as sediments, plant-associated bacteria, or marine environments, where detailed information is currently missing.

Despite the fact that the information about Gemmatimonadota is accumulating, still, little is known about their metabolism and, thus, their environmental role. They usually form only a small fraction of the bacterial community, with relative abundances at around 1%. These low numbers in the environment could relate to their slower growth, which is often associated with the ability to withstand stressful conditions [124]. Members of this group are able to survive in extreme environments, such as saline soils [87], soils in Antarctica [43,46], hypersaline soda lakes [19], or deep-sea sediments [64,99]. This could signify that they are K-strategists with less active metabolisms and resistance against environmental stresses at the cost of lower growth rates. On the other hand, it cannot be excluded that the slow growth rates of cultured species could be the result of suboptimal media choices and/or the need for specific compounds, as specific growth rates of >2d^−1^ of freshwater Gemmatimonadota were reported from a manipulation experiment [125]. The observed relationship with algae and cyanobacteria [18] could be another reason for their low numbers and the difficulties in culturing them. Such relationships could result in a patchy distribution and local predominance in specific microhabitats [83]. Further research should focus on their importance in food webs and biogeochemical cycles. Special attention should be paid to the key biogeochemical processes, such as photo(hetero)trophy, carbon assimilation, phosphorus acquisition, or nitrogen and sulfur metabolism. Potential roles in cycling of nutrients were already highlighted in discoveries of members capable of anoxygenic photosynthesis [15,16], phototrophic MAGs with a possible capacity for carbon fixation [19,121], and a member with potential N_2_O reduction capabilities [24]. Additional laboratory experiments with cultured species are necessary in order to elucidate their metabolic properties and physiology. These experiments would be complementary to bioinformatic methods that enable the metabolic potential to be studied in many uncultured organisms by using the available metagenomes. The analysis of metagenome-assembled genomes offers an immense amount of information for studying this interesting but difficult-to-culture bacterial group.

## Data Availability

Not applicable.

## Supplementary Materials

The following are available online at https://www.mdpi.com/article/10.3390/microorganisms10010151/s1, Table S1: Accession numbers of sequences used for the phylogenetic tree in Figure 5.
